# Baicalein-corrected gut microbiota may underlie the amelioration of memory and cognitive deficits in APP/PS1 mice

**DOI:** 10.3389/fphar.2023.1132857

**Published:** 2023-03-30

**Authors:** Jing Shi, Jie Chen, Xinyun Xie, Yuanyuan Li, Wenjing Ye, Jianbiao Yao, Xiangnan Zhang, Tianyuan Zhang, Jianqing Gao

**Affiliations:** ^1^ School of Pharmaceutical Sciences, Institute of Materia Medica, Hangzhou Medical College, Hangzhou, China; ^2^ College of Pharmaceutical Sciences, Zhejiang University, Hangzhou, China; ^3^ Key Laboratory of Neuropsychiatric Drug Research of Zhejiang Province, Hangzhou, China; ^4^ Zhejiang CONBA Pharmaceutical Co Ltd, Hangzhou, China

**Keywords:** Baicalein, Alzheimer’s disease (AD), memory and cognition, metagenomics, metabonomics

## Abstract

**Background:** Baicalein is an active ingredient extracted from the root of S. baicalensis Georgi, which exhibits cardiovascular protection, anti-inflammatory, and anti-microbial properties. Our previous study showed that chronic treatment of Baicalein ameliorated cognitive dysfunction in a mouse model of Alzheimer's disease (AD). However, it remains unknown whether Baicalein ameliorates cognitive deficits in AD mouse models by altering gut microbiota and its metabolites.

**Methods:** Behavioral tests, metagenomic and untargeted metabolomics analyses were used to evaluate the effects of Baicalein on the APP/PS1 mice.

**Results:** Our research showed that treatment of Baicalein for 2 weeks ameliorated cognition and memory in a dose-dependent manner, as indicated by the significant increases in the Discrimination index and Number of crossings and decrease in latency to the previous platform location in 8-month of age APP/PS1 mice in novel object recognition and water maze tests. The metagenomic analysis showed the abundance of the dominant phyla in all groups, including Bacteroidetes (14.59%–67.02%) and Firmicutes (20.19%–61.39%). LEfSe analysis of metagenomics identified three species such as s__Roseburia_sp_1XD42_69, s__Muribaculaceae_bacterium_Isolate_104_HZI, s__Muribaculaceae_bacterium_Isolate_110_HZI as Baicalein-treated potential biomarkers. Metabolite analysis revealed the increment of metabolites, including glutamate, thymine and hexanoyl-CoA.

**Conclusion:** The effects of Baicalein on memory and cognition may relate to the metabolism of nucleotides, lipids and glucose.

## Introduction

Alzheimer’s disease (AD) is the most common and progressive neurodegenerative disease that seriously affects patients’ thinking ability, cognition, and ability to perform daily activities (Gu et al., 2016). Beta-amyloid (Aβ) plaques, neurofibrillary tangles, neuronal atrophy, and death are the major pathological features of AD. However, the underlying mechanisms of whether these pathological changes induce cognitive impairment and dementia have not been fully elucidated. Moreover, no therapies for the treatment of AD have been approved for more than a decade. Currently available treatments can only temporarily improve some symptoms but not halt or reverse disease symptoms and progression. Several clinical trials of single-target treatments have recently failed to modify cognitive and memory deficits (Cummings et al., 2019). The complex pathophysiology of AD requires multi-target therapies, such as those correcting neuronal function and systemic abnormalities in metabolism (Varma et al., 2018), rather than monotherapy, which prompted us to develop effective disease-modifying treatments involving drugs that may specifically bind to multiple targets, such as those in the metabolism in both central and peripheral nervous systems (CNS and PS), to ensure clinical effectiveness and reduce toxicity.

The root of Scutellaria baicalensis Georgi, a classic compatible component in the decoction of herbal medicine, may have such anti-AD effects. Baicalein is an active ingredient extracted from the root of S. baicalensis Georgi, which exhibits cardiovascular protection, anti-inflammatory, and anti-microbial properties (Gu et al., 2016; Yan et al., 2018). Recent studies have reported that Baicalein was effective in the treatment of neurodegeneration, such as AD and Parkinson’s disease (PD) (Zhang et al., 2017; Sonawane et al., 2021). Increasing evidence suggested that Aβ peptide accumulation and subsequent neuronal deficits were linked to systemic abnormalities in metabolism (Wei et al., 2014; Varma et al., 2018). The clinical investigations revealed that AD patients exhibit differences in gut microbial diversity compared with age-matched controls (Kim et al., 2020). Another study suggested altered intestinal microbiota and marked intestinal inflammation in AD mice, associated with forming amyloid plaques and neurofibrillary tangles (Kim et al., 2014). Moreover, the transfer of healthy microbiota to AD mice significantly decreased amyloid plaques and tau hyperphosphorylation, further supporting the pivotal role of gut metabolism in AD development. However, it is unclear how global gut metabolism interventions are related to the expression of AD symptoms after Baicalein treatment. Therefore, we proposed that the changes in the gut microbiota and its metabolites may be a potential mechanism of AD pathogenesis, and Baicalein may improve AD cognitive function by affecting the gut microbiota.

## Materials and methods

### Animals

Transgenic male APP/PS1 mice (8-month-old, weight 22–30 g) were purchased from Beijing Huafukang Bioscience CO, LTD. (HFK, Beijing, China), and age-matched male wild-type C57BL/6 mice were obtained from the Animal Center of Hangzhou Medical College (Hangzhou, China). They were acclimatized to the laboratory environment for 5–7 days before the experiment. At the animal center, the mice were housed in a barrier system with specific pathogen-free conditions, controlled temperature (25°C ± 1 °C), and a 12-h light/dark cycle. The animals had free access to water and food. The experimental procedures were performed as per the National Institutes of Health Guide for Animal experimentation and the European Communities Council Directive of 24 November 1986 (86/609/EEC). All procedures were approved by the Care and Use of Laboratory Animals Committee of Hangzhou Medical College.

### Drugs and treatment

**FIGURE 1:**
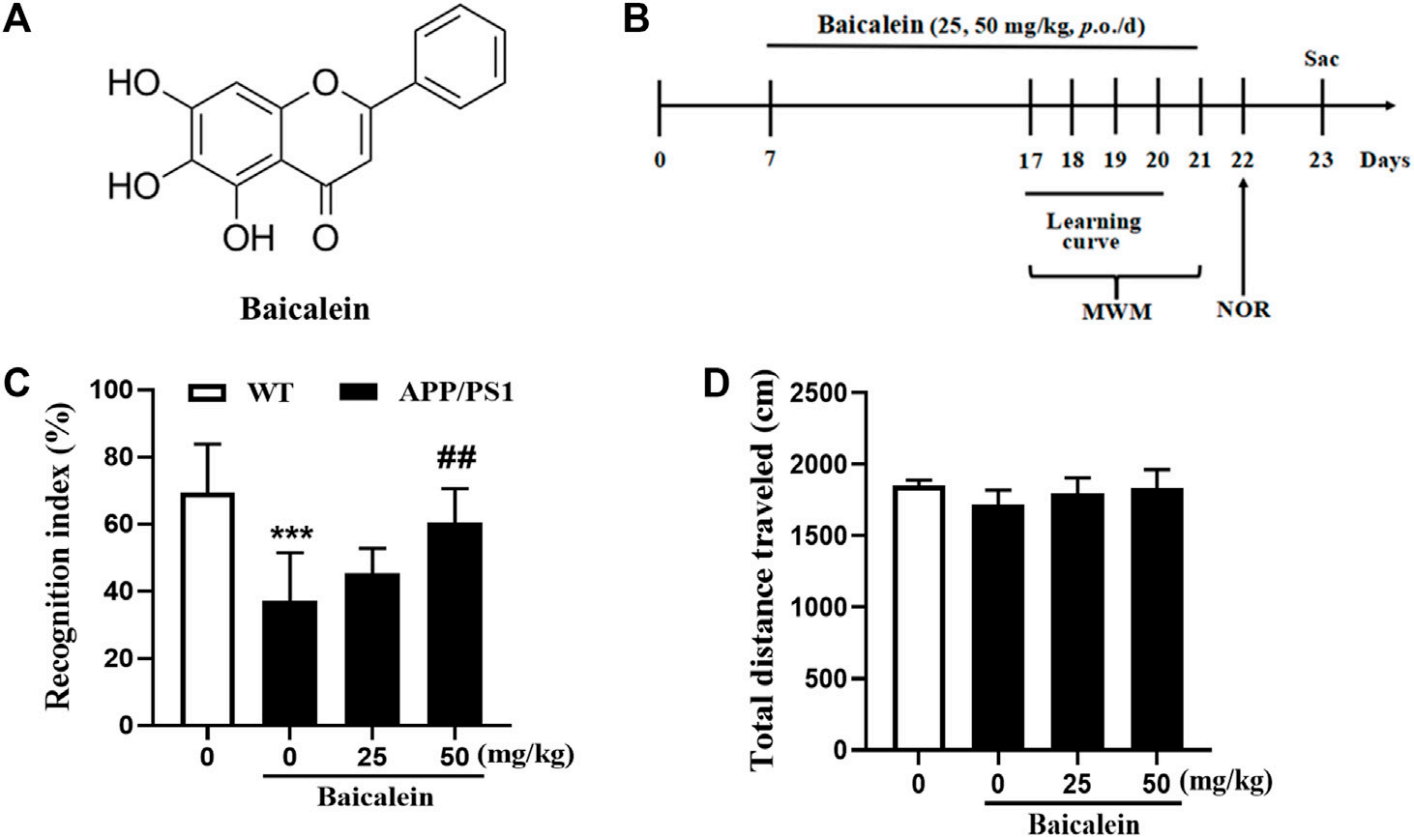
Baicalein improved cognitive function of APP/PS1 mice in the novel object recognition test. **(A)** The chemical structure of Baicalein. **(B)** Treatment timeline and test order for mice treated with different doses of Baicalein. **(C)** The recognition index was shown by the ratio of time spent exploring the novel object to the familiar one. **(D)** The total distance traveled in different groups of mice. All values were expressed as Mean ± SEM (*n* = 6). ^***^ *p <* 0.001 vs wild type (WT) mice; ^##^ *p <* 0.01 vs APP/PS1 mice.

Baicalein was purchased from Sigma-Aldrich Chemical Corporation (St. Louis, MO, United States ) and was dissolved in dimethyl sulfoxide (DMSO). The concentration did not exceed 0.1% of the total volume in the working solution. The chemical structure of Baicalein is shown in Figure 1A. Baicalein at 25 mg/kg and 50 mg/kg (determined from our pilot experiments) was continuously administered *via* gavage (*i. g., once a day*) for 14 days according to previous studies (Gu et al., 2016; Shi et al., 2021).

### Morris water maze

The Morris Water Maze (MWM) test was conducted following the previous study with minor modifications (Xu et al., 2015). A water maze apparatus was put in a well-illuminated room with a circular, plastic pool (1.5 m diameter × 0.5 m high) filled with water at room temperature (22°C ± 1 °C). There was a platform 1 cm below the water surface in the maze. An automatic image tracking system was connected to a behavior analysis system for monitoring learning and memory behaviors. The formal experiments included two sessions, e.g., the acquisition trials and probe trials. In the acquisition trial, the mice were put in the center of four quadrants facing the wall individually. They learned to become familiar with the environment. The acquisition trial was conducted four times per day at 20 min intervals for 4 days. The time to stay on the platform within 60 s was recorded as latency. The probe trial was conducted 4 days after training. The swimming speed, the total distance, and the path moved by the mice were recorded. The number of entries in the target quadrant, the percentage (%) of time spent in the target quadrant, and latency to the target quadrant where the platform was previously located were determined 24 h after training.

### Novel object recognition (NOR) test

The novel object recognition (NOR) test was performed to measure the object memory recognition ability of mice that reflected the degree of scattered memory loss in mice similar to the symptoms of AD (Xu et al., 2015). A transparent, open cube box (40 × 40 × 45 cm) was selected for the experiment, which was divided into three stages: habituation, training, and probe trials. In the habituation trial, all the mice were allowed to explore the apparatus for 5 min freely. The training trial was conducted by putting an individual mouse in the center of the chamber. The device contained two fixed and odorless identical objects located in 2 diagonal corners. In the test trial, the mice were placed back in the center of the same chamber for 5 min; however, one of the familiar objects was replaced with a novel object. The ratio of time spent on new objects to the total time for exploring both novel and old objects was calculated as the recognition index (RI).

### Metagenomic sample preparation and analysis

The stool samples were stored at −80 °C after collection. DNA from stool samples was extracted using the Stool DNA Kit (Omega Bio-Tek, Inc, United States ) according to the manufacturer’s instructions. The purity and quality of the genomic DNA were assessed on 1% agarose gels and Qubit detection.

DNA was sheared to 300-bp fragments by using the Covaris ultrasonic crusher. To prepare the sequencing library, the fragments were subjected to end repair, tailing, and ligation of Illumina-compatible adapters. DNA sequencing libraries were deep sequenced on the Illumina HiSeq platform at Allwegene Company (Beijing). After the run, image analysis, base calling, and error estimation were performed using Illumina Analysis Pipeline Version 2.6. The quality control of the raw data was performed using the Trimmomatic method (Bolger et al., 2014), which included the removal of the adapter sequence and low-quality reads. The reads having the adapter sequence, N (uncertain base) ratio greater than 1%, and the content of low-quality base (Q ≤ 20) greater than 50% were filtered. The reads whose length was still less than 150 bp were removed after quality control.

High-quality sequences were compared with the NR database and classified into different taxonomic groups using the diamond (Buchfink et al., 2015). MEGAHIT (Li et al., 2015) was used to assemble the sequencing data, and the contigs below 500 bp were removed. Contigs were annotated with Prodigal software (Hyatt et al., 2012) to predict open reading frames, and CD-HIT software (Li et al., 2001) was used to construct the non-redundant gene set. Bowtie (Langmead et al., 2009) was used to compare the sequencing data with the non-redundant gene set, and the information on gene abundance in different samples was obtained. The gene function was annotated by searching against the functional annotation databases, namely, KEGG, COG/KOG, GO, CARD, and CAZyme.

### Metabolomic and metabolite analyses

The metabolites were detected using UHPLC-QE-MS (UHPLC-QE-MS) at Allwegene Company (Beijing, China). Statistical analyses included data standardization, principal component analysis (PCA), OPLS-DA analysis, Student’s t-test, and screening and enrichment analysis of differential metabolites. Differential metabolites were screed based on the following criteria: *p*-value of the Student’s t-test <0.05 and the VIP value >1. The personalized analysis included KEGG analysis of differential metabolites and metabolic pathway analysis. Differential metabolites were mapped using KEGG, PubChem, and other metabolite databases, and pathways involved in all differential metabolites were identified. The differential metabolites were further analyzed for metabolic pathways. Through a comprehensive analysis (including enrichment and topological analyses) of the pathway where the differential metabolites were located, further pathway screening was performed to identify the key pathway with the strongest correlation with differential metabolites.

### Statistical analysis

Statistical analysis was performed using R. All data are expressed as the mean ± standard deviation (SD). Differences among multiple groups were analyzed by one-way analysis of variance (ANOVA), with *post hoc* comparisons of *Dunnett’s* test. Two groups were compared using the *t*-test. The interpretation of the symbols (* and #) for the different group comparisons are shown in the Figure legends. The Spearman algorithm was used to determine the correlation between metagenomics and metabolomics. R and Python were used to draw graphics. Statistical significance was set at *p* < 0.05.

## Results

### The Baicalein ameliorated recognition memory deficits in NOR test in APP/PS1 mice

The drug administration protocol is given in Figure 1B. The NOR test was performed to assess episodic-like memory. In this study, 8-month-old APP/PS1 mice exhibited significant memory impairment, as evidenced by a decreased RI 24 h after the training session compared with age-matched wild-type mice (Figure 1C, *p <* 0.001). One-way ANOVA revealed that Baicalein improved memory retention and retrieval in a dose-dependent manner, as indicated by the increased RI compared with that of the vehicle-treated APP/PS1 mice [Figure 1C, F (3,20) = 8.749, *p <* 0.001]. As shown in Figure 1C, a high dose (50 mg/kg) of Baicalein administration significantly increased the exploration ability of the mice to the novel object (*p <* 0.01), but not changed in low dose group (25 mg/kg), which indicated that 50 mg/kg of Baicalein better improve episodic-like memory of APP/PS1 mice. No significant change was observed among the groups in the total distance traveled (Figure 1D), indicating that the performance differences did not result from the changes in overall activity.

### Baicalein ameliorated spatial memory deficits in the MWM test in APP/PS1 mice

**FIGURE 2:**
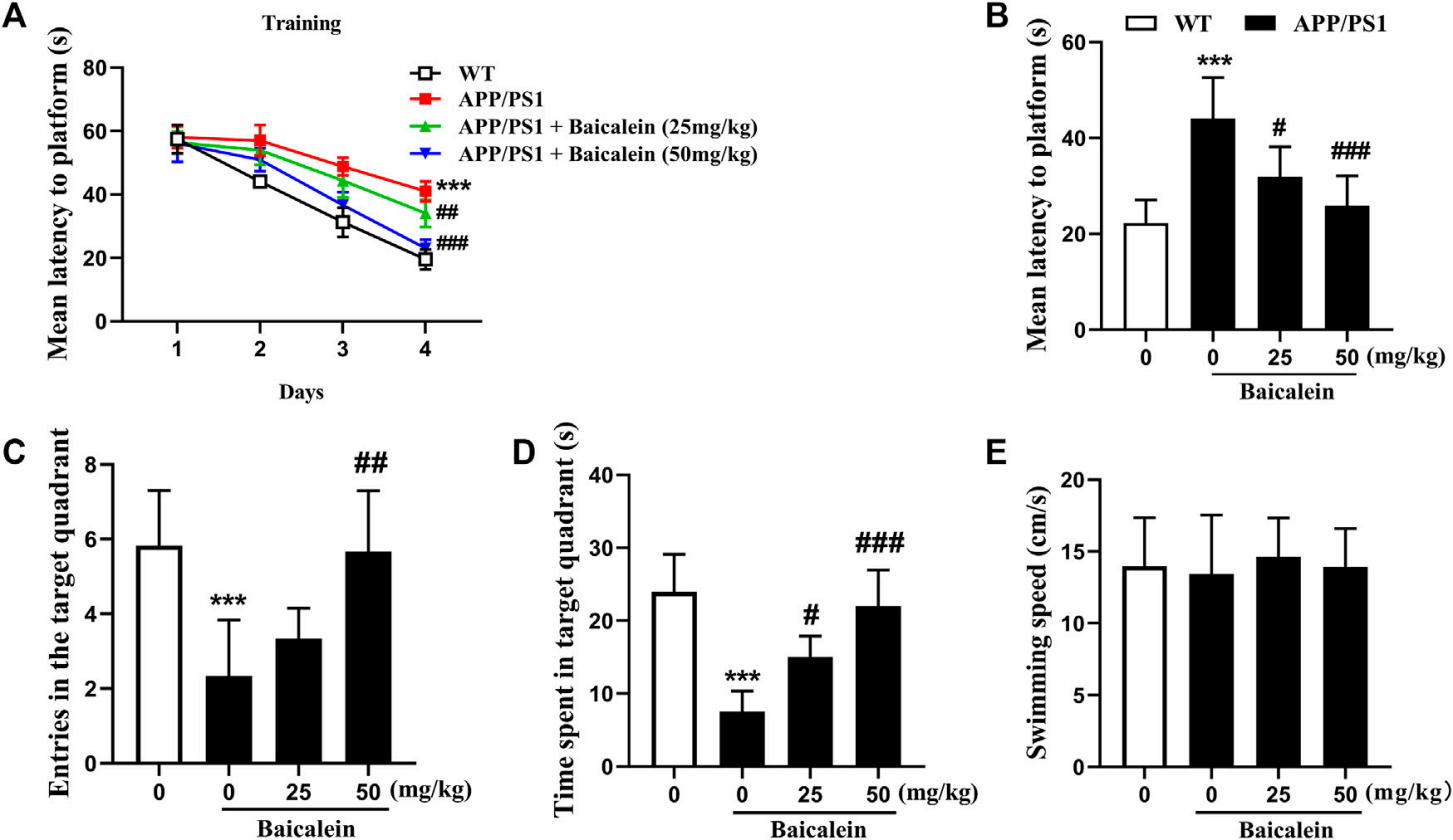
Baicalein improved memory acquisition and retention of APP/PS1 in the Morris water maze (MWM) test. The learning curve **(A)**, Mean latency to the platform **(B)**, Frequency of entering the target quadrant **(C)**, Duration of time spent in the target quadrant **(D)**, and the swimming speed **(E)** of different groups of mice in the MWM test. All values were expressed as Mean ± SEM (*n* = 6). ^***^ *p <* 0.001 vs WT mice; ^#^ *p <* 0.05, ^##^ *p <* 0.01, ^###^ *p <* 0.001 vs APP/PS1 mice.

In the MWM test, 8-month-old APP/PS1 mice showed longer latency to reach the platform on day 4 (Figure 2A, *p* < 0.001) and longer mean latency to the platform (Figure 2B, *p* < 0.001) as compared with the age-matched control group. This impairment was rescued by the administration of Baicalein at 25 mg/kg (*p <* 0.05) and 50 mg/kg (*p <* 0.001) intraperitoneal in a dose-dependent manner. On Day 5 in MWM, APP/PS1 mice had poorer performance than non-dementia control, as evidenced by a decreased number of platforms crossing (Figure 2C, *p <* 0.001), and reduced time in the target quadrant (Figure 2D, *p <* 0.001). Baicalein at 25 mg/kg and 50 mg/kg ameliorated memory retention also in a dose-dependent manner, as shown by the increased number of platforms crossing (Figure 2B, *p <* 0.001) and increased time spent in the target quadrant (Figure 2D, *p <* 0.001). Furthermore, Baicalein exhibited an overall enhancing effect on the entries to the platform at a higher dose of 50 mg/kg (Figure 2C, *p <* 0.01; Figure 2D, *p <* 0.001) in the 24 h probe trial. No significant changes in swimming speed were observed among the groups (Figure 2E).

### Baicalein treatment reversed the changes in intestinal microbial diversity in APP/PS1 mice

**FIGURE 3:**
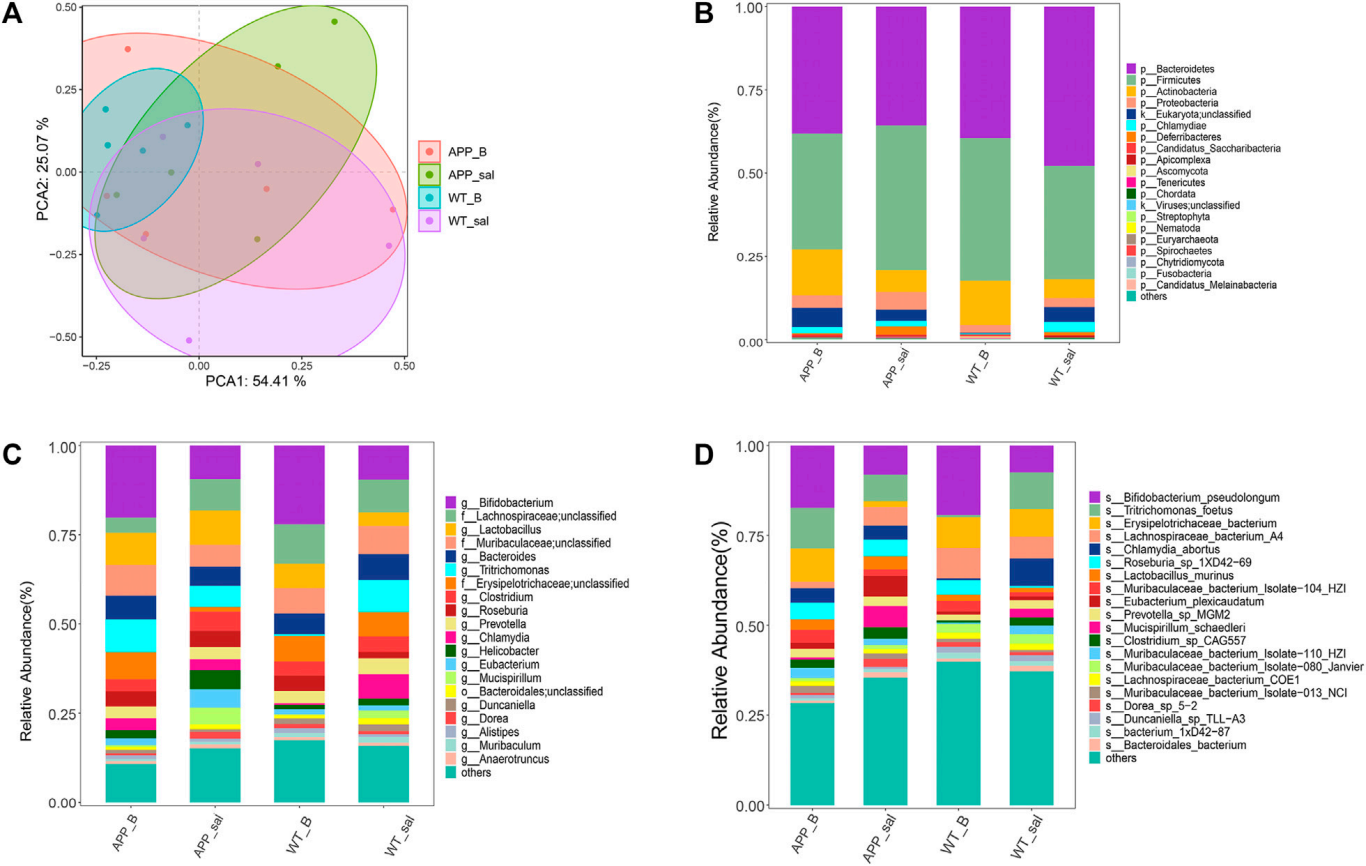
Microflora alteration in the WT and APP/PS1 mice treated with Baicalein. **(A)** Comparison of microbiota based on species composition by principal component analysis (PCA) in WT and APP/PS1 mice treated with saline (WT_sal and APP_sal) or Baicalein (WT_B and APP_B). **(B–D)** Composition of species in different taxa. WT_sal, APP_sal, WT_B and APP_B were colored at the phylum level on a bar graph, and genus and species levels were presented as bar graphs in all groups.

PCA was performed to assess the differences between APP/PS1 mice and WT controls. To investigate the effect of Baicalein treatment on intestinal microflora changes related to the pathogenesis of enteritis, the wildtype-Baicalein treated (WT_B) group was used to eliminate background differences between the mice. PCA results revealed significant differences between APP/PS1-saline treated (APP_sal) and APP/PS1-Baicalein treated (APP_B) microbial communities. The species composition of wildtype-saline (WT_sal), WT_B, and APP_sal groups was similar (Figure 3A). In addition, we annotated the species and analyzed the species composition histogram; the results revealed that the species abundance and composition of APP_sal were different from that of WT_sal and APP_B. The dominant phylum of all taxa, including p_Bacteroidetes (14.59%–67.02%) and p_Firmicutes (20.19%–61.39%) (Figure 3B). The abundance of p_Firmicutes and p_Proteobacteria was the highest in the APP_sal group and Species abundance was reduced in APP/PS1 mice when they were treated with Baicalein. The abundances of these two species in the APP_B group and WT_sal group were the same. These results indicated that the normal levels of the above-mentioned parameters could be restored after Baicalein treatment. By analyzing the data at the genus level, we observed that the abundance of g_*Lactobacillus*, g_Bifidobacterium, g_Tritrichomonas, and g_*Clostridium* were relatively high in APP_sal groups. However, the relative abundance of these species was increased in APP/PS1 mice when they were treated with Baicalein (Figure 3C). In addition, the relative abundance of s_Eubacterium Plexicaudatum, s_Lachnospiraceae bacterium 3-2, s_Dorea SP 5-2, and s_Ruminococcus_sp_1xD21-23 in the APP_sal group was significantly higher than that in the WT_sal group; while the abundance of the aforementioned species in the APP_B group was significantly low. These findings suggested that Baicalein treatment blocked the AD-induced increase in the abundance of these species (Figure 3D).

### Comparative analysis of intestinal microbiota in different groups of mice

**FIGURE 4:**
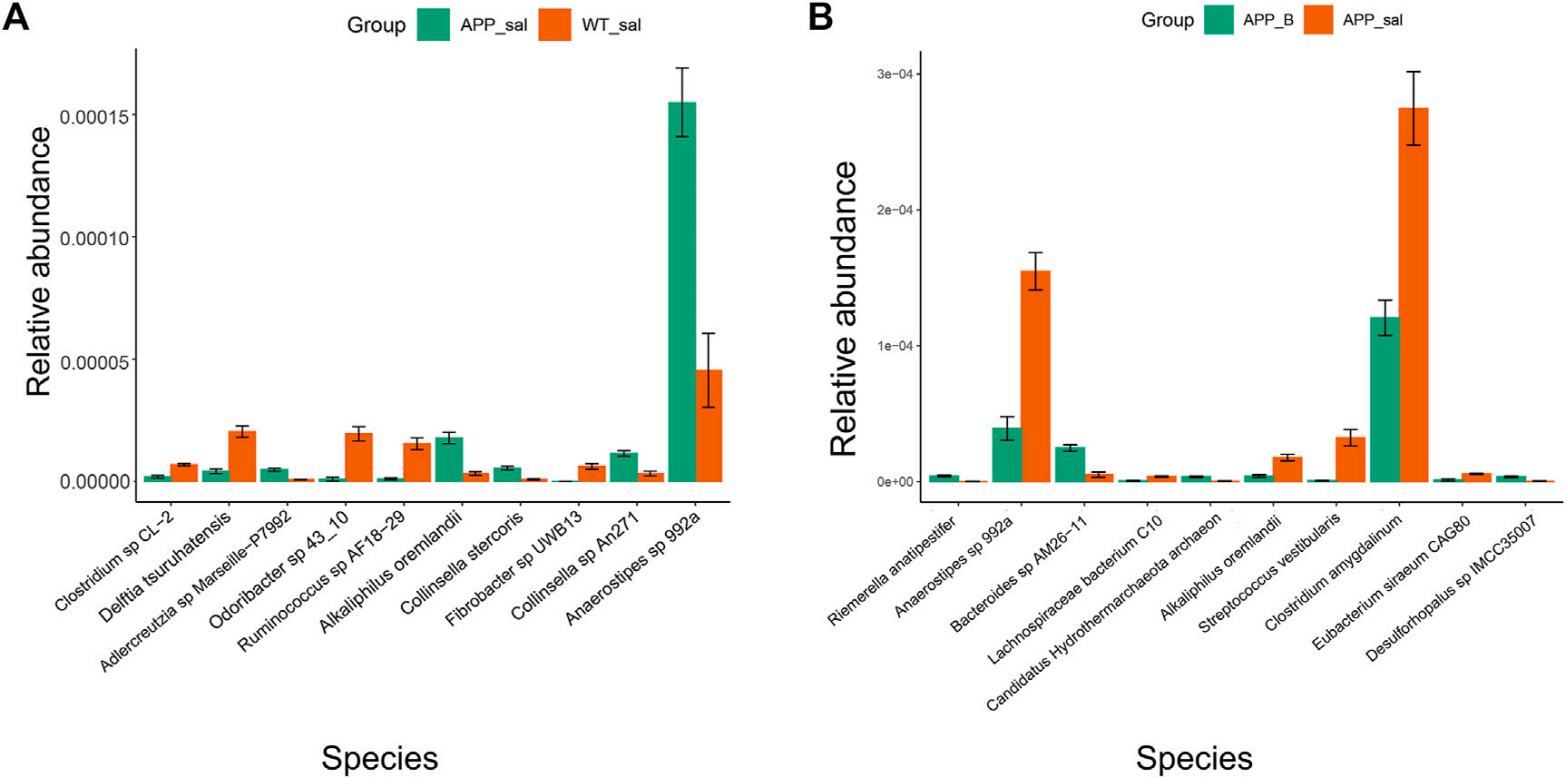
Metaststes analysis shows differential species. Pairwise comparisons of species with significant differences were obtained by Metastats analyses in APP_sal vs. WT_sal group **(A)**, and APP_B vs. APP_sal **(B)**. *p* values <0.05 were considered significant.

According to the results of Metastats, the abundance of s_Fibrobacter_sp_UWB13 (*p <* 0.05), s_Ruminococcus_sp_AF18-29 (*p <* 0.05), and s_Odoribacter_sp_43_10 (*p <* 0.05) in the APP_sal group was significantly lower than that in the WT_sal group. However, the APP_sal and APP_B groups exhibited no significant difference in the abundance of these species (Figure 4A). The abundance of s_Anaerostipes_sp_992a (*p <* 0.05) and s_*Streptococcus*_vestibularis (*p <* 0.05) in the APP_sal group was significantly higher than that in the APP_B groups. The species abundances in the WT_sal and APP_B groups were similar at the species level. These results suggested that Baicalein treatment can reverse the abundance of these species (Figure 4B).

### Biomarkers of the gut microbiome in different groups of mice

**FIGURE 5:**
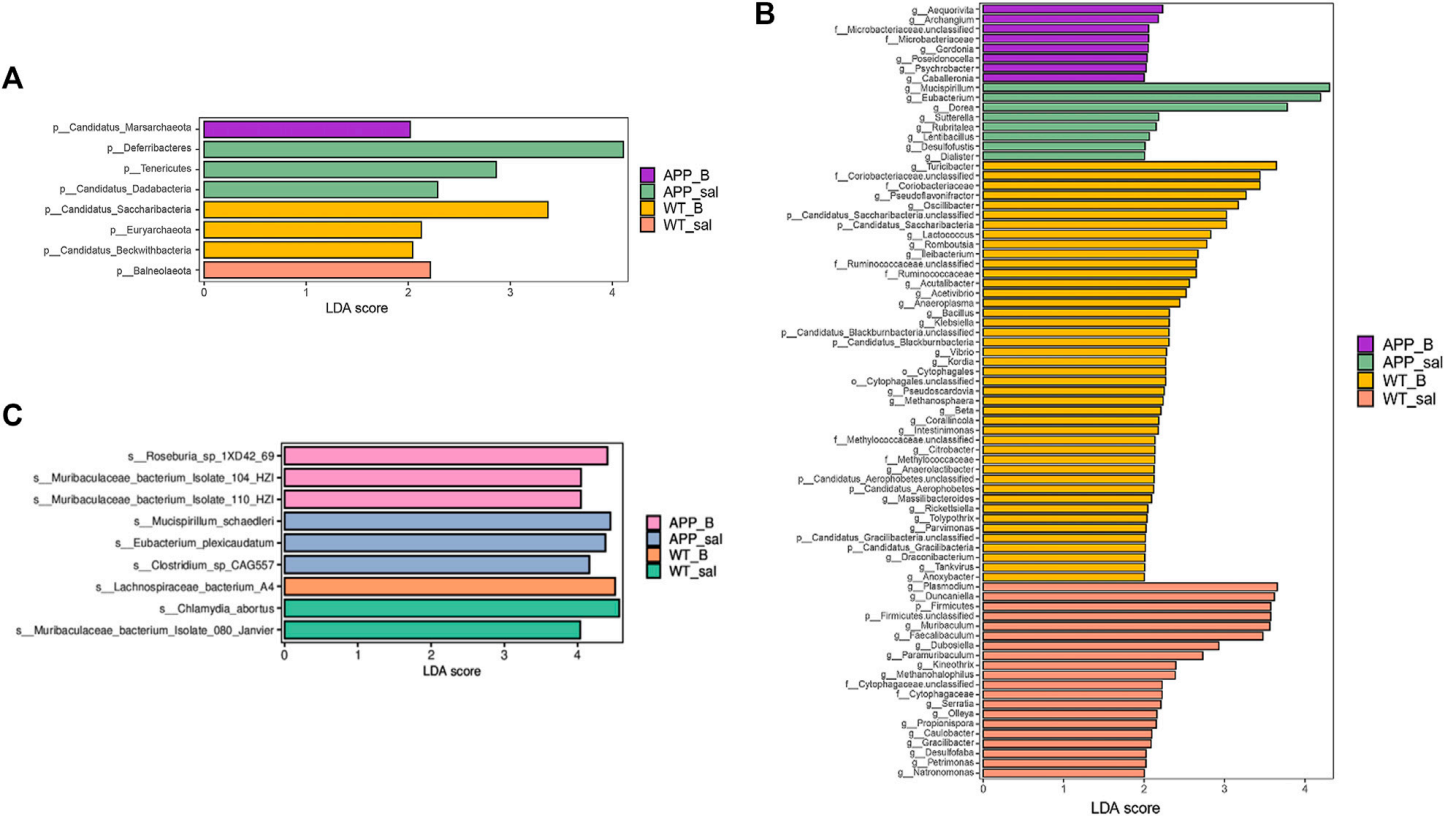
Linear discriminant analysis (LDA) effect size (LEfSe) analysis of the key biomarkers in different groups. **(A-C)** LEfSe analysis of APP_B vs. APP_sal microbiota. LDA scores of APP_sal and APP_B (log10 > ± 2) groups indicated greater abundance at the phylum level. LDA scores of APP_sal and APP_B (log10 > ± 3) groups indicated greater abundance at the genus level. LDA score of APP_sal compared with APP_B (log10) > ± 4 was more abundant at the species level.

Linear discriminant analysis (LDA) of effect size (LEfSe) in combination with the statistical analysis was used to screen key biomarkers after treatment with Baicalein. The LDA scores of APP_sal and APP_B (log10 > ±2) groups showed greater abundance at the phylum level. The LDA scores of APP_sal and APP_B (log10 > ±3) groups showed greater abundance at the genus level. LDA scores of APP_sal and APP_B (log10> ±4) groups indicated greater abundance at the species level. Metagenomic results revealed that p_Deferribacteres can be considered as a specific biomarker of the APP_sal group at the phylum level, whereas p_candidatus_saccharibacteria can be considered as a specific biomarker of the WT_B group (Figure 5A). At the genus level, three specific biomarkers in the APP_sal group were identified. The WT_B group had seven specific biomarkers, and the WT_sal group had six specific biomarkers (Figure 5B). At the species level, three specific biomarkers in the APP_sal group were identified, including s__*Clostridium*_sp_CAG557, s_Mucispirillum_schaedleri, and s__Eubacterium_plexicaudatum. In contrast, the WT_sal group had two specific biomarkers, including s__Muribaculaceae_bacterium_Isolate_080_Janvier and s__*Chlamydia*_abortus. The APP_B group had three specific biomarkers, including s__Roseburia_sp_1XD42_69, s__Muribaculaceae_bacterium_Isolate_104_HZI, and s__Muribaculaceae_bacterium_Isolate_110_HZI. Finally, s_Lachnospiraceae_bacterium_A4 is a unique biomarker of the WT_B group (Figure 5C).

### Baicalein treatment changed the intestinal metabolites of APP/PS1 mice

**FIGURE 6:**
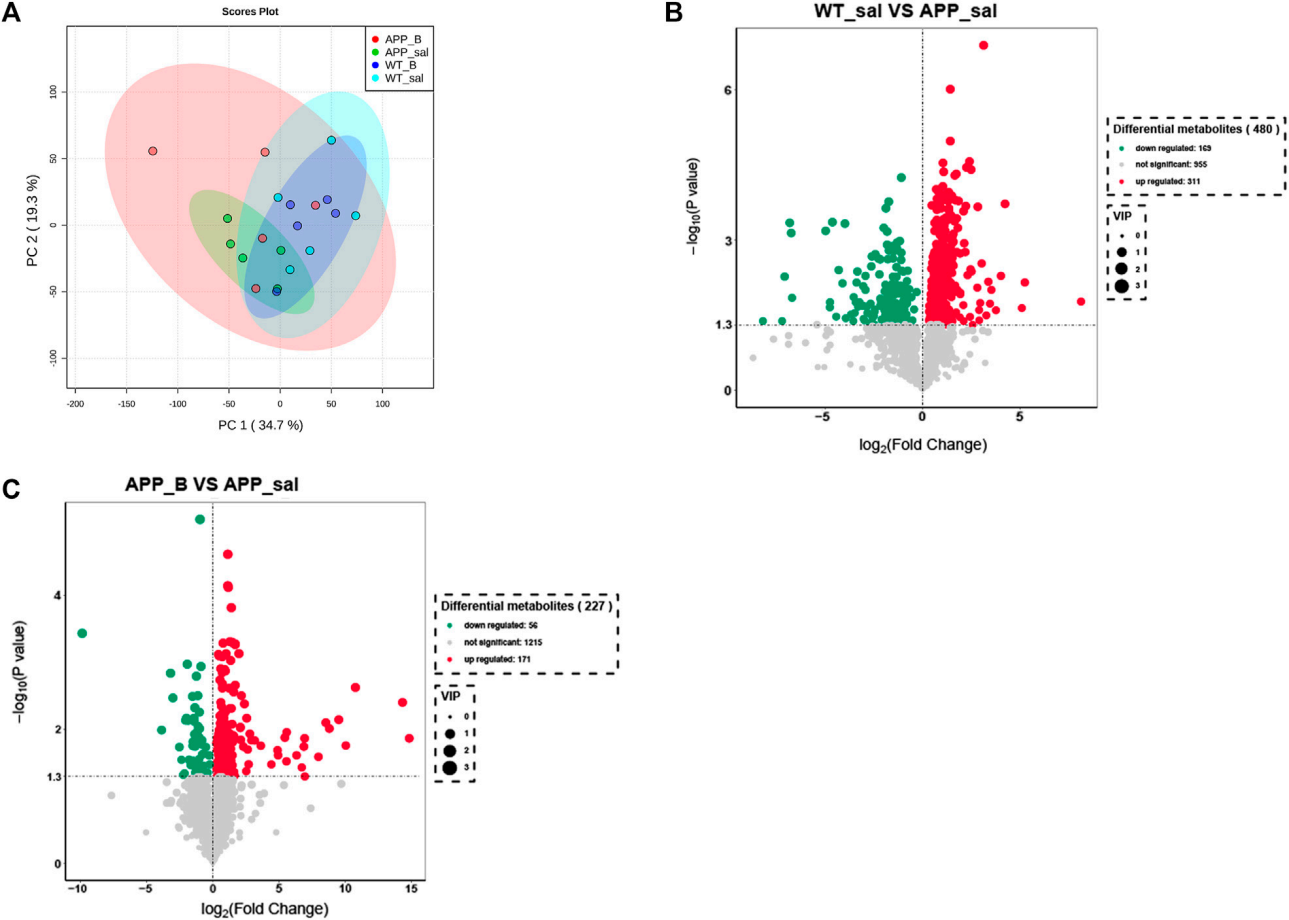
Baicalein treatment changed intestinal metabolites in the APP/PS1 mice. **(A)** Partial least squares discriminant analysis (PLS-DA) score plots of metabolites in different groups. Volcano map of metabolite changes in the WT_sal vs. and APP_sal groups **(B)**, and APP_B vs. APP_sal groups **(C)**. Downregulated metabolites are marked in green and Upregulated metabolites are marked in red.

**FIGURE 7:**
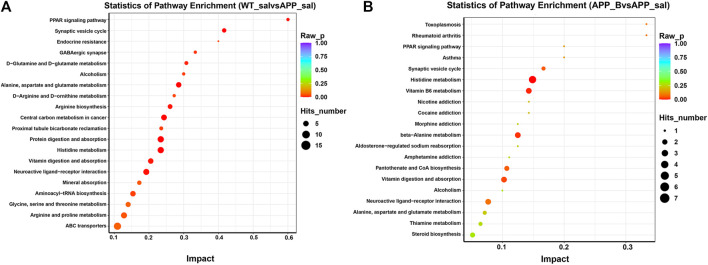
KEGG pathway enrichment in different groups. The top 20 metabolic pathways in the comparison combinations were analyzed according to impact factors in the WT_sal vs and APP_sal groups **(A)**, and APP_B vs APP_sal groups **(B)**. The number of metabolites enriched in each term is shown as the circle size, and the *p*-value is shown as different colors.

To determine the effect of Baicalein on the alterations in gut metabolites associated with AD, we used UHPLC-QE-MS and PCA cluster analysis to conduct metabonomic studies. The metabolic changes of the APP_sal group relative to the WT_sal or APP_B group were further determined. All samples were analyzed with a 95% confidence interval (Figure 6A). The upregulation of various metabolites was displayed in the form of a volcano to screen differential metabolites. Overall, 480 metabolites were significantly different between the WT_sal and APP_sal groups, among which 169 were downregulated and 311 were upregulated (Figure 6B). These metabolites were enriched in 146 metabolic pathways, particularly focusing on the metabolism of alanine, aspartic acid, and glutamate; the central carbon in cancer metabolism; protein digestion and absorption; histidine metabolism; neuroactive ligand-receptor interaction; as well as other metabolic pathways (Figure 7A). 227 metabolites were observed, with significant differences between the APP_B and APP_sal groups. Furthermore, 56 downregulated differential metabolites and 171 upregulated differential metabolites were enriched in 72 metabolic pathways (Figure 6C), which Focus on histidine metabolism and microbial B6 metabolism, beta-alanine metabolism, and other pathways, respectively (Figure 7B).

### Association analyses of metagenomics and metabonomics

To determine the correlation between the metagenome of AD mice and various metabolites, we analyzed the microbial and host metabolites of the intestinal flora of the APP/PS1 mice. The intersections of APP_sal and APP_B, APP_B and WT_sal, and WT_ Sal and WT_B were used to screen 25 metabolites with the following criteria: FC values <0.25 and >4, and *p* values <0.05.

Totally 6 biomarkers in group APP_sal and APP_B at the species level were identified using the LEfSe analysis with the following criteria: LDA>4, which include_Roseburia_sp_1XD42_69, s_Muribaculaceae_bacterium_Isolate_104_HZI, s_*Clostridium*_ sp_CAG557, s_Mucispirillum_schaedleri, s_Muribaculaceae_bacterium_Isolate_110_HZI, s_Eubacterium_plexicaudatum. The correlation analysis was conducted for these 25 metabolites.

### Correlation analysis between different metabolites and species

**FIGURE 8:**
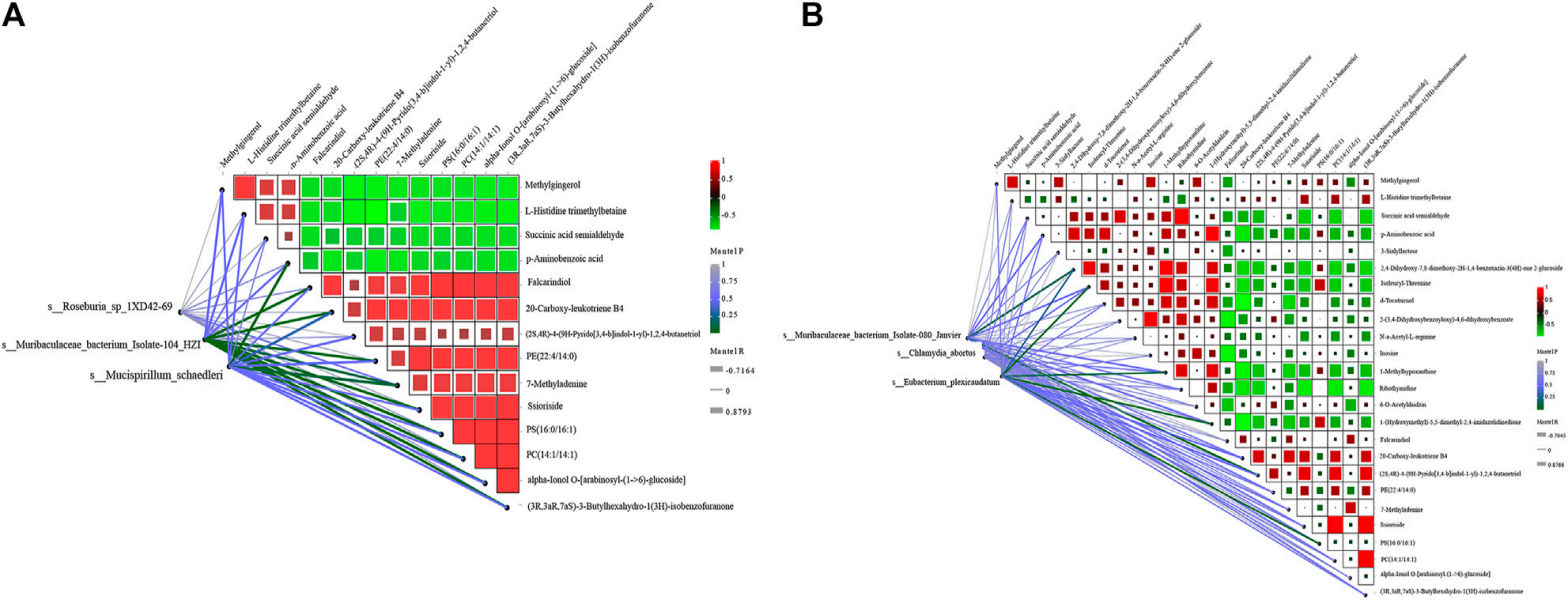
Correlation analysis between different metabolites and species. **(A)** Correlation analysis between three key biomarkers was obtained by screening APP_sal and APP_B and differential metabolites. **(B)** Correlation analysis between the three key biomarkers was obtained by screening APP_sal and WT_sal and the differential metabolites. The magnitude of the correlation between metabolites was indicated by the color of the block. The correlation between species and metabolites was determined using the Mantel test. The greener the line, the lower the *p*-value.

According to the LDA value, two biomarkers related to group APP_sal, namely, s_Mucispirillum_schaedleri and s_Eubacterium_plexicaudatum. Two biomarkers related to group APP_B, namely, s_Roseburia_sp_1XD42_69 and s_Muribaculaceae_bacterium_Isolate_104_ HZI, and two biomarkers related to group WT_sal, namely, s_*Chlamydia*_abortus and s_ Muribaculaceae_bacterium_Isolate_080_Janvier were screened for mantel test. In the APP_B and APP_sal groups, the correlation analysis revealed that L-histidine trimethylbetaine was positively correlated with methylgingerol. Moreover, both Ssioriside and Falcarindiol exhibited significant positive correlations with multiple metabolites, and the correlation coefficient was greater than 0.9. Methylgingerol was significantly negatively correlated with multiple metabolites. The correlation coefficients were all greater than 0.9. According to the Mantel test results, s_Muribaculaceae_bacterium_isolate-104_HZI with PS (16:0/16:1), PC (14:1/14:1), alpha-Ionol O-[arabinosyl-(1->6)-glucoside] were strongly correlated with each other, and the correlation coefficient was >0.85. A significant positive correlation was observed between s_Mucispirillum_schaedleri and p-Aminobenzoic acid, and the correlation coefficient was >0.8 (Figure 8A).

In the APP_sal and WT_sal groups, the correlation analysis revealed that Ssioriside and PC (14:1/14:1) (3R,3aR, 7aS)-3-butylhexahydro-1(3H)-isobenzofuranone, PC (14:1/14:1) were significantly positively correlated with (3R,3aR, 7aS)-3-butylhexahydro-1(3H)-isobenzofuranone, succinic acid semialdehyde, and ribothymidine. The correlation coefficients were all greater than 0.9. Furthermore, a significant negative correlation was observed between d-tocotrienol and 20-carboxy-leukotriene B4, with a correlation coefficient greater than 0.9. Mantel test revealed that the s_*Chlamydia*_abortus, s_Eubacterium_plexicaudatum, s_Muribaculaceae_bacterium_Isolate_ 080_Janvier screened were significantly positively correlated with different metabolites. A significant positive correlation was observed between s_Muribaculaceae_bacterium_isolates-080_janvier (2S, 4R)-4-(9H-pyrido [3,4-b]indul-1-yl)-1,2,4-butanetriol, s_Eubacterium_ plexicaudatum, and isoleucyl-threonine (Figure 8B).

### Functional association of metabolites with APP_B or APP_sal biomarkers

The results of functional annotation of metabolites associated with key biomarker genes of APP_B or APP_sal were enriched. KEGG annotation results revealed that these two key biomarkers were involved in purine metabolism, histidine metabolism, tyrosine metabolism, and nicotinate and nicotinamide metabolism. PE (14:0/15:0), succinic acid semialdehyde, and inosine were correlated with APP_sal biomarkers (s_*Clostridium*_sp_CAG557). Both APP_sal and APP_B biomarkers encoded several enzymes in the succinic acid semiphosphate metabolism pathway (KO00760). However, L-histidine trimethylbetaine and succinic acid semialdehyde were highly correlated with the biomarker of APP_B (s_Roseburia sp. 1XD42-69) (Table 1).

**TABLE 1:** Functional annotations of associated metabolites with genes of biomarkers of APP_sal or APP_B mice.

Name	KO_id	Gene_name	EC	Metabolic pathways	PATH_ko	Metabolites
s__Roseburia sp. 1XD42-69	K02501	hisH	EC:4.3.2.10	Histidine metabolism	ko00340	L-Histidine trimethylbetaine
	K00013	hisD	EC:1.1.1.23			
	K01496	hisI	EC:3.5.4.19			
	K01733	thrC	EC:4.2.3.1	Vitamin B6 metabolism	ko00750	Succinic acid semialdehyde
	K12410	npdA	EC:2.3.1.286	Nicotinate and nicotinamide metabolism	ko00760	Succinic acid semialdehyde
	K00858	ppnK, NADK	EC:2.7.1.23			
	K00767	nadC, QPRT	EC:2.4.2.19			
	K00278	nadB	EC:1.4.3.16			
s__*Clostridium*_sp_CAG557	K00969	nadD	EC:2.7.7.18	Nicotinate and nicotinamide metabolism	ko00760	Succinic acid semialdehyde
	K03742	pncC	EC:3.5.1.42			
	K00995	pgsA, PGS1	EC:2.7.8.5	Glycerophospholipid metabolism	ko00564	PE (14:0/15:0)
	K08591	plsY	EC:2.3.1.275			
	K00655	plsC	EC:2.3.1.51			
	K00951	relA	EC:2.7.6.5	Purine metabolism	ko00230	Inosine

Among the biomarkers of APP_sal, the genes mainly involving metabolic pathways were nadD, pncC, plsY, and plsC. These genes particularly encode nicotinate-nucleotide adenylyltransferase, nicotinamide-nucleotide amidase, acyl phosphate: glycerol-3-phosphate acyltransferase, 1-acyl-Sn-glycerol-3-phosphate acyltransferase, *etc.* Among the biomarkers of APP_B, the genes mainly involving metabolic pathways were hisH, hisD, hisI, and thrC, which mainly encode imidazole glycerol-phosphate synthase subunit HisH, histidinol dehydrogenase, phosphoribosyl-AMP cyclohydrolase, and threonine synthase pathways.

## Discussion

In the present study, chronic Baicalein treatment for 14 days significantly reversed cognitive deficits in 8-month-old APP/PS1 mice. The composition and metabolites of gut microbiota were altered in the APP/PS1 mice, and Baicalein changed the gut microbiota and subsequently corrected the metabolism of AD mice to that of controls. Modulating the gut microbiota through various diets and beneficial microbiota interventions may serve as an AD therapy. Alterations in gut microbes led to changes in the metabolites by influencing the amino acid, taurine, hypotaurine, glutathione, and histidine metabolism pathways. Thus, Baicalein administration could be a novel strategy to treat AD *via* reshaping the gut microbiome and metabolites.

Many herbal remedies are now gathering more attention as complementary and alternative interventions and are important sources for developing drug candidates for AD. More and more studies are investigating the use of various medicinal plants and their principal phytochemicals for the treatment of AD. One example of such herbal medicine is Scutellaria baicalensis Georgi. Baicalein is the main bioactive ingredient in this herbal plant, with anti-inflammatory, antioxidant, and cognitive-enhancing properties (Brinza et al., 2021; Gregory et al., 2021). Our previous study showed that chronic treatment of Baicalein ameliorates cognitive dysfunction in the novel object recognition and Morris water maze tests in the Aβ-treated mouse model of AD (Shi et al., 2021). However, whether Baicalein treatment could alter gut metabolism remains unknown. In the present study, we extended our previous study and found that Baicalein treatment for 2 weeks improved cognition in a dose-dependent manner, as evidenced by the significantly increased time to explore the novel object in 8-month of age APP/PS1 mice in the novel object recognition test. Moreover, Baicalein decreased mean latency to the previous platform location and increased the entries to the target quadrant and time spent in the target quadrant in APP/PS1 mice, further supporting the memory-enhancing effects of Baicalein.

The earliest clinical symptoms of Alzheimer’s disease are changes in episodic memory, compromised judgment, and orientation. These early findings correlate with synapse loss in the hippocampus induced by amyloid-beta (Aβ) 1–42 plaque burden (Gu et al., 2016). The synaptotoxic Aβ1-42 can be modeled in mice by microinjection of Aβ1-42 oligomers bilaterally into the hippocampus, and Baicalein protected mice against Aβ1-42 induced AD-like cognitive impairment in our previous study (Shi et al., 2021). In the present study, the “gold standard” transgenic mouse Alzheimer’s models, e.g., APP/PS1 mouse model, were used, in which plaque accumulation was age-dependent. We found that chronic treatment of Baicalein improved sporadic and spatial memory in novel object recognition and Morris water maze tests in these AD mice, further suggesting that Baicalein exhibits memory enhancing effects.

Anti-AD therapeutics remain challenging due to the limited understanding of disease mechanisms. Previous studies suggested that amyloid plaques and neurofibrillary tangles are essential features of AD (Briggs et al., 2016). Recent studies point to lifestyle, diet, environmental, and genetic factors contributing to AD pathological development. Increasing evidence suggests that the microbiota is transformed prior to the onset of AD, and the gut microbial alteration is closely related to the early stage of AD (Li et al., 2019). Diet plays a critical role in determining intestinal microbiota composition, impacting several major metabolic pathways, and may link to immune responses (Anand and Mande, 2018). Some studies found that lifestyle modifications such as exercise and nutritional intervention specifically affect the anti-AD treatment effect (Scheltens et al., 2016). These findings are consistent with the clinical investigations that suggest that *Helicobacter pylori* infection is a significant risk factor for the pathological development of neurological diseases such as AD (Wang et al., 2015; Beydoun et al., 2020). *Helicobacter pylori* can cross the blood-brain barrier (BBB) in the disease state. Some studies suggest that *H. pylori* are responsible for neuroinflammation and the subsequent amyloid beta accumulation and BBB disruption, which resultantly interacts with gut metabolites and leads to neuronal damage in AD mouse models (van de Haar et al., 2015; Contaldi et al., 2017). Microbes are also found to affect the peripheral immune response, as evidenced by the increase in pro-inflammatory cytokines such as IL-6 and IL-1beta in the intestine, which causes the structural change of the BBB, leading to the loss of BBB integrity (Cryan and Dinan, 2012). Moreover, intestinal inflammation expedites the accumulation of neurofibrillary tangles and amyloid plaques, further supporting the crucial role of altered gut metabolism in AD pathology (Minter et al., 2017; Marizzoni et al., 2020). The anti-inflammatory effects of Baicalin are significant for AD cognitive improvement, but the inflammatory parameters were not shown in the present study and will be explored in future studies.

The subsequent metagenomic sequencing results showed that Baicalein treatment significantly changed intestinal metabolites in these 8-month APP/PS1 mice. These changes were involved in metabolisms of alanine, aspartic acid, and glutamate, the central carbon in cancer metabolism, protein digestion and absorption, histidine metabolism, and neuroactive ligand-receptor interaction. After LEfSe analysis of metabolic pathways related to these metabolisms, six biomarkers about APP_sal and APP_B were significantly positively correlated with different metabolites, for example, six biomarkers with the largest LDA values associated with APP_sal, APP_B, and WT_sal. These results suggested that s_Roseburia_sp_1XD42-69, s_Muribaculaceae_ bacterium_Isolate-104_HZI, s_*Chlamydia*_abortus, s_Mucispirillum_schaedleri, s_ Eubacterium_plexicaudatum, and s_Muribaculaceae_bacterium_Isolate_080_Janvier were significantly positively correlated with different metabolites; methylgingerol was significantly negatively correlated with multiple metabolites. Obviously, these metabolites have a functional association with the Baicalein treatment. KEGG annotation results revealed that two key biomarkers (s_Roseburia sp. 1XD42-69 and s_*Clostridium*_sp_CAG557) were involved in purine metabolism, histidine metabolism, tyrosine metabolism, and nicotinate and nicotinamide metabolism. These results indicated that Baicalein treatment could significantly improve the cognitive ability of mice may *via* the gut microbiome and metabolite dysfunction. Further experiments are needed to determine how Baicalein improves memory by regulating intestinal microbiota directly or indirectly.

Glutamate is an essential excitatory neurotransmitter in neurons, and abnormal increases in glutamate may lead to cell death (Zhang et al., 2016). Glutamatergic neurons play an important role in cognitive function, which is closely associated with the progression of AD, and anti-glutamate can be used as a treatment for AD (Zott et al., 2019). Our study showed that baicalein treatment affects glutamate metabolism, suggesting that it may improve cognitive function in APP/PS1 mice *via* the anti-glutamate pathway. Niacin includes two vitamers, nicotinic acid and nicotinamide, giving rise to the coenzymatic forms nicotinamide adenine dinucleotide (NAD) and nicotinamide adenine dinucleotide phosphate (NADP), which are required for oxidative reactions and crucial for energy production. NAD and NADP regulate biological functions, including gene expression, cell cycle progression, DNA repair and cell death (Gasperi et al., 2019). Previous studies show an inverse association between AD and niacin intakes, and dietary niacin may protect against AD and age-related cognitive decline (Morris et al., 2004). Nicotinamide also can counteract amyloid toxicity by reducing the expression of AD-related genes (Wang et al., 2016). The abnormal Nicotinate and nicotinamide metabolism in our study may reveal the possible effects of Baicalein on AD treatment. Studies of glycerophospholipid composition have shown that levels of phosphatidylcholine (PC), phosphatidylethanolamine (PE) and phosphatidylinositol (PI) are significantly reduced in different regions of neural membranes in AD patients compared to age-matched control human brains (Igarashi et al., 2011). An increase in lipid mediators levels may contribute to abnormal signal transduction processes and neurodegeneration in AD (Frisardi et al., 2011). Lower concentrations of glycerophospholipids were associated with greater severity of both amyloid and neurofibrillary pathology (Varma et al., 2018). Due to the critical role of glycerophospholipids, baicalin may regulate glycerophospholipid metabolism by affecting the gut microbiota, thereby improving AD cognitive impairment.

## Conclusion

Baicalein exhibited an overall enhancing memory consolidation and retrieval in novel object recognition and Morris water maze tests. The present study also demonstrated how Baicalein improved learning and memory by regulating the gut microbiome and metabolite dysfunction in APP/PS1 mice. These results provide a valuable reference for using gut microbiota as a diagnostic biomarker and therapeutic target in clinical studies of AD.

## Data Availability

The datasets presented in this study can be found in online repositories. The names of the repository/repositories and accession number(s) can be found in the article/Supplementary Material.
